# Association between ZJU index and concurrent hypertension and hyperuricemia among adults undergoing health examinations in Wuhu, China: a cross-sectional study

**DOI:** 10.3389/fcvm.2026.1974735

**Published:** 2026-10-12

**Authors:** Errui Song, Huan Wu, Tao Wu, Xinru Wang, Lu Zhou, Fan Su, Catherine Adaeze Ezeokafor, Yue Wu, Xinyu Ma, Tong Wang, Yufeng Wen

**Affiliations:** 1 School of Public Health, Wannan Medical College, Wuhu, Anhui, China; 2 School of Laboratory Medicine, Wannan Medical College, Wuhu, Anhui, China; 3 School of Public Health, Anhui Medical University, Hefei, Anhui, China

**Keywords:** hypertension, hyperuricemia, insulin resistance, subgroup analysis, ZJU index

## Abstract

**Background:**

The Zhejiang University (ZJU) Index, integrating BMI, fasting plasma glucose, triglycerides, and liver enzymes, has been established as a predictor for non-alcoholic fatty liver disease (NAFLD). Given the strong interconnections among NAFLD, hypertension (HTN), and hyperuricemia (HUA), we hypothesized that the ZJU index might be associated with their comorbid state (HTN-HUA). However, no comprehensive study has yet fully elucidated this association.

**Methods:**

This cross-sectional investigation analyzed data from individuals undergoing health examinations at the First Affiliated Hospital of Wannan Medical College in Wuhu, China, from 2017 to 2023, enrolling 468,558 participants. The association between the ZJU index and HTN-HUA was assessed using restricted cubic spline (RCS) analysis. Logistic regression models explored associations of the ZJU index with HTN-HUA, HTN, and HUA. Receiver operating characteristic (ROC) analyses compared the discrimination of ZJU alone, clinical covariates without ZJU, and clinical covariates plus ZJU.

**Results:**

Among 468,558 participants, 44,225 (9.4%) had HTN-HUA, 162,858 (34.8%) had HTN, and 90,521 (19.3%) had HUA. RCS analysis demonstrated a non-linear relationship between the ZJU index and HTN-HUA. After adjustment for covariates, the ZJU index showed significant positive associations with HTN-HUA, HTN, and HUA. Compared to the lowest quartile, the highest ZJU quartile had increased odds of 7.01 (95% CI: 6.72–7.31) for HTN-HUA, 5.66 (95% CI: 5.53–5.79) for HTN, and 4.01 (95% CI: 3.90–4.11) for HUA. For HTN-HUA, the AUC was 0.725 (95% CI: 0.723–0.727) for ZJU alone, 0.785 (95% CI: 0.783–0.787) for the clinical covariate model, and 0.827 (95% CI: 0.825–0.828) for the clinical covariate model with ZJU.

**Conclusion:**

Elevated ZJU index values were significantly associated with higher odds of prevalent HTN-HUA, suggesting its potential value as a marker of the metabolic profile associated with concurrent hypertension and hyperuricemia among adults undergoing health examinations.

## Introduction

Hypertension (HTN) and hyperuricemia (HUA) have shown a marked increase in prevalence worldwide over recent decades (1, 2). Hypertension is an established risk factor for cardiovascular disease (3, 4). It is also associated with hypertension-mediated organ damage and premature mortality (5). Hyperuricemia has been associated with coronary heart disease morbidity and mortality (6). Their widespread occurrence has also placed a considerable burden on healthcare systems globally (7, 8). The close relationship between HUA and HTN has been consistently documented across numerous population-based studies (9, 10). It has been estimated that approximately 25%–40% of individuals with hypertension also present with HUA (11). Moreover, the coexistence of HTN and HUA confers a substantially greater risk of cardiovascular disease than either condition alone (12). Accordingly, the European Society of Hypertension has designated serum urate a major determinant in the stratification of cardiovascular risk among patients with hypertension (5). Therefore, early identification of individuals with concurrent hypertension and hyperuricemia (HTN-HUA) is of considerable clinical importance.

Extensive epidemiological evidence has consistently demonstrated that unhealthy behavioral factors—such as tobacco use, heavy alcohol consumption, and obesity—contribute substantially to the development of both HUA and HTN (13–15). When evaluating these metabolic abnormalities, conventional obesity-related indicators, particularly body mass index (BMI), have important limitations. Although BMI reflects general body size, it does not directly quantify abdominal, visceral, or ectopic adiposity (16). Visceral and ectopic fat are closely associated with cardiometabolic disease (17). A recently proposed waist-adjusted mass index also illustrates an approach that incorporates waist circumference into anthropometric assessment (18). Therefore, individuals with a normal BMI may nevertheless have adverse metabolic characteristics, a phenotype commonly described as metabolically obese normal weight (MONW) (19). Accordingly, a composite index that integrates anthropometric and metabolic information may complement BMI in characterizing metabolic risk.

Formulated by combining BMI with fasting plasma glucose (FPG), triglycerides (TG), alanine aminotransferase (ALT), and aspartate aminotransferase (AST), the ZJU index is a composite index integrating anthropometric and biochemical measures (20, 21). These components reflect aspects of adiposity, glycemia, lipid metabolism, and liver-enzyme activity. A prospective cohort study has also investigated the ZJU index in relation to gestational diabetes mellitus (22).

Importantly, the clinical relevance of the ZJU index has progressively extended beyond its original application in hepatic steatosis. Subsequent evidence demonstrated that the ZJU index was also associated with insulin resistance in the Chinese general population, suggesting that this composite measure may capture broader metabolic dysfunction rather than hepatic steatosis alone (23). More recently, population-based studies have reported significant associations of the ZJU index with hypertension, further supporting its potential utility as a marker of systemic cardiometabolic risk (24).

Accumulating evidence suggests that hypertension and hyperuricemia share several metabolic and vascular pathways, including insulin resistance, visceral adiposity, oxidative stress, endothelial dysfunction, and abnormal renal handling of sodium and uric acid (12, 23). Insulin resistance may contribute to both elevated blood pressure and hyperuricemia through alterations in renal urate handling and sodium retention (23). In addition, elevated uric acid has been implicated in endothelial dysfunction through oxidative stress and reduced nitric oxide bioavailability, while experimental and clinical evidence also suggests a potential role of uric acid in the pathogenesis of hypertension (23, 25). These interconnected processes suggest that a composite metabolic indicator integrating adiposity, glucose metabolism, triglyceride metabolism, and hepatic metabolic function may capture, at least in part, the shared metabolic milieu underlying the coexistence of hypertension and hyperuricemia.

However, evidence regarding the relationship between the ZJU index and hyperuricemia remains limited, particularly with respect to the coexistence of hypertension and hyperuricemia among adults undergoing health examinations. A recent study among adults with type 2 diabetes reported a positive association between the ZJU index and hyperuricemia, providing initial evidence for a potential link between this composite metabolic index and uric acid metabolism (26). However, whether the ZJU index is associated with the combined phenotype of hypertension and hyperuricemia in a large population undergoing health examinations remains insufficiently characterized.

This cross-sectional study examined the association between the ZJU index and prevalent HTN-HUA among adults undergoing health examinations in Wuhu, China. We also evaluated the apparent discrimination of ZJU alone and its addition to a clinical covariate model within the same analytical sample.

## Methods and materials

### Study participants

This cross-sectional survey used health checkup records of adults aged 18–90 years from 2017 to 2023 provided by the First Affiliated Hospital of Wannan Medical College in Wuhu, China. Inclusion criteria required participants to be ≥18 years old with complete anthropometric measurements and informed consent. The original source dataset, before preliminary cleaning, contained 652,044 records. After excluding 72,892 records with missing ZJU values, 579,152 remained. Among these records, 109,896 had missing GGT values; overall, 110,594 records with missing data for age, sex, smoking, drinking, GGT, LDL, or HTN-HUA were excluded, leaving 468,558 unique participants (255,715 men and 212,843 women) in the complete-case sample used for all analyses (Figure 1). Variable-specific missingness among the 579,152 records with non-missing ZJU was as follows: age, 3 (0.0005%); smoking, 950 (0.1640%); drinking, 1,019 (0.1759%); GGT, 109,896 (18.9753%); and LDL, 86 (0.0148%).

**Figure 1:**
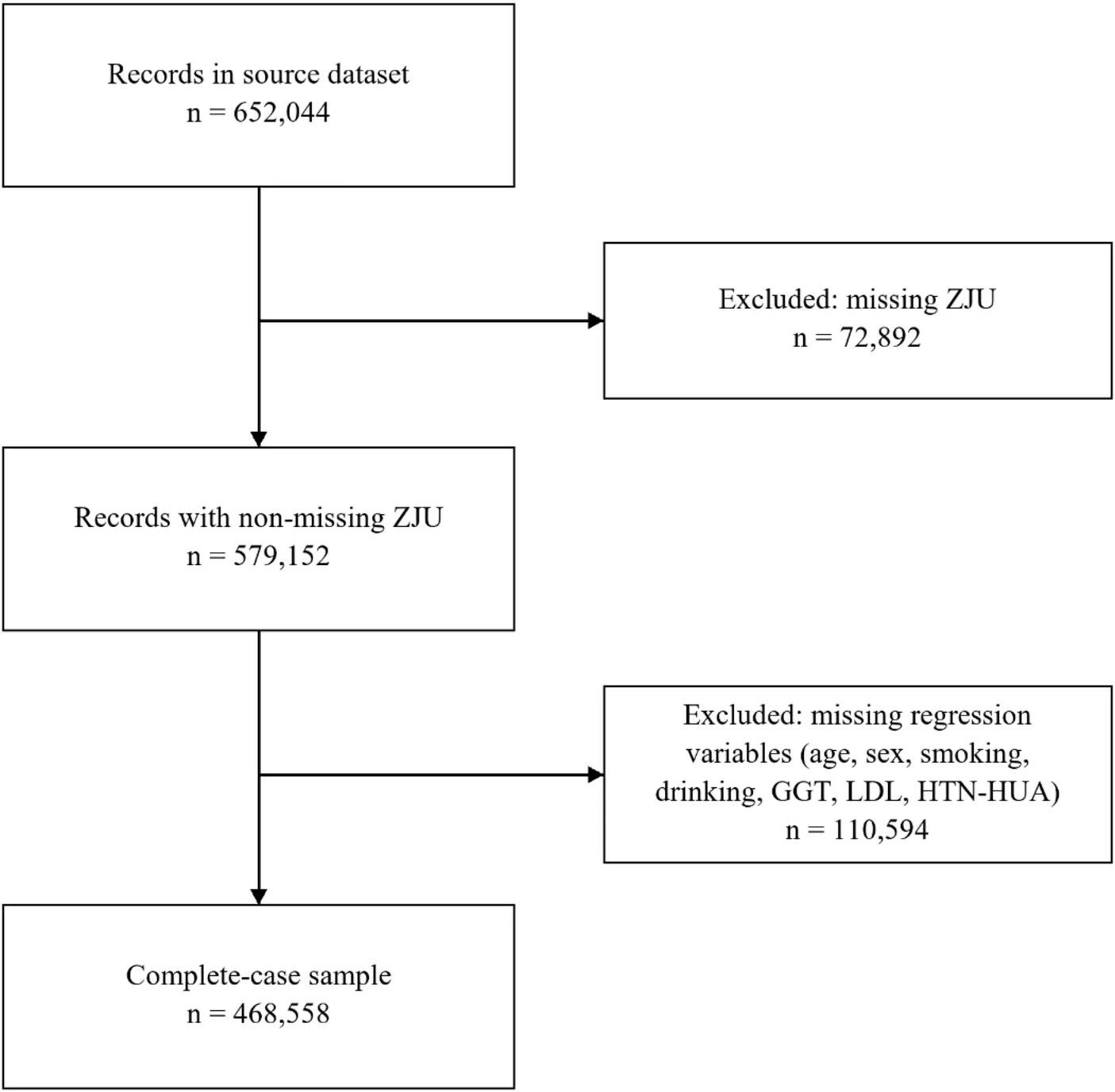
Participant disposition.

The study protocol adhered to the ethical principles of the Declaration of Helsinki and was approved by the Institutional Review Board of Wannan Medical College (approval No. 2024-LS-007). Written informed consent was obtained from all participants at the time of the original health examinations. The 2024 ethics approval covered the retrospective use and analysis of these previously collected data.

### Anthropometric and laboratory measurements

Standardized questionnaires were used to gather basic demographic information, including age, sex, smoking habits, and alcohol use. Smoking and alcohol consumption were assessed by questionnaire and recorded as ordinal variables coded 0–3, based on self-reported daily cigarette consumption and alcohol intake, respectively. Anthropometric data were obtained from participants dressed in light clothing without footwear. Height and weight were measured to the nearest 0.1 cm and 0.1 kg, respectively. BMI was calculated as weight (kg) divided by height squared (m^2^) and retained to two decimal places.

Professional technicians used standardized equipment to measure resting blood pressure in the right arm after at least 5 min of rest. Two measurements were obtained, and their average was used to record systolic blood pressure (SBP) and diastolic blood pressure (DBP). The available documentation did not permit confirmation of participant position, cuff selection, or the interval between blood pressure measurements. The method used to ascertain medication use could not be verified. Fasting venous blood specimens were obtained and assayed within 24 h of collection using automated biochemistry analyzers. Biochemical analyses included serum uric acid (SUA), total cholesterol (TC), triglycerides (TG), low- and high-density lipoprotein cholesterol (LDL and HDL), non-HDL cholesterol (computed as TC minus HDL), fasting plasma glucose (FPG), alanine and aspartate aminotransferases (ALT and AST), blood urea nitrogen (BUN), and creatinine (SCR). All procedures were conducted by trained professionals, with results cross-verified for accuracy.

### Diagnostic criteria

1. **Diabetes:** FPG ≥7.0 mmol/L (27).
2. **Hyperuricemia (HUA):** SUA >420 μmol/L (men) or >360 μmol/L (women) (28, 29), or current use of urate-lowering medication.
3. **Hypertension (HTN):** Systolic blood pressure ≥140 mmHg and/or diastolic blood pressure ≥90 mmHg, or ongoing antihypertensive drug therapy (30).
4. **ZJU Index:** The ZJU index was computed as the sum of BMI (kg/m^2^), FPG (mmol/L), TG (mmol/L), 3 × (ALT/AST), plus 2 for women (20).
5. **HTN-HUA:** In the present study, HTN-HUA was defined as the concomitant presence of both HTN and HUA. For the analyses of HTN and HUA as individual outcomes, HTN and HUA were defined independently according to the diagnostic criteria described above, regardless of whether the other condition was present.

### Statistical analysis

We conducted all data analyses with R (version 4.4.3). For each outcome, logistic models incorporated ZJU as a restricted cubic spline with four knots at the 5th, 35th, 65th, and 95th percentiles (28.48, 32.98, 36.50, and 43.78). Models adjusted for age, sex, smoking, drinking, GGT, and LDL; age, GGT, and LDL were continuous, whereas sex, smoking, and drinking were categorical. Odds ratios were referenced to the sample median ZJU (34.66). Likelihood-ratio tests compared the spline model with the covariate-only model for the overall association (3 degrees of freedom), and with a linear-ZJU model for nonlinearity (2 degrees of freedom). All analyses used the same 468,558 complete cases. Unique personal identifiers were used to prevent duplicate inclusion, and the analytical dataset contained one record per individual. The 468,558 participants with complete data for ZJU, HTN-HUA, age, sex, smoking, drinking, GGT, and LDL were divided into ZJU quartiles Q1–Q4 using the quartiles calculated within this complete-case sample. Table 1 summarizes their baseline characteristics. Continuous summaries and comparisons used available observations for each variable. Continuous variables were presented as mean ± SD (x¯ ± s) and compared via the Kruskal–Wallis H procedure; while categorical data have been presented in the form of counts (proportions) and examined with the chi-squared (*χ*^2^) analysis. Multivariable logistic regression models have been constructed to evaluate the associations of ZJU index quartile categories (independent variable) with the outcomes for HTN-HUA, HTN, and HUA (dependent variables). Model 1 contained no covariate adjustments. Model 2 controlled for age and sex. Model 3 additionally controlled for age, sex, smoking, drinking, GGT, and LDL. ROC analyses compared the ZJU score alone, a logistic model containing age, sex, smoking, drinking, GGT, and LDL without ZJU, and the same model with a continuous linear ZJU term. The latter two ROC curves used fitted probabilities. AUC confidence intervals and paired comparisons used the DeLong approach. Thresholds maximized sensitivity + specificity−1 (Youden index), with scores or probabilities at or above the threshold classified as positive. Thresholds are on the ZJU scale for ZJU alone and the probability scale for multivariable models. All ROC estimates represent apparent performance in the fitting sample; no validation or calibration analysis was performed. Subgroup models used the Model 3 covariates, omitting the stratifying variable (age was omitted in age-stratified models). Age strata were <40, 40–65, and >65 years. Interaction was tested by likelihood-ratio comparison of models with and without ZJU-quartile-by-stratum interaction terms. Odds-ratio confidence intervals used the Wald method. Age- and sex-stratified ROC analyses evaluated ZJU alone. A two-sided *P* < 0.05 was considered statistically significant. The quartile cut points (displayed to two decimal places) were 31.83, 34.66, and 37.99; full-precision values were used for grouping, and intervals were right-closed, with the minimum included in Q1.

**Table 1:** Baseline features of participants according to ZJU index quartiles.

Variable	Overall (*n* = 468,558)	Q1 (*n* = 117,140)	Q2 (*n* = 117,139)	Q3 (*n* = 117,139)	Q4 (*n* = 117,140)	*p*-value
Age (years)	45.31 ± 13.10	41.96 ± 13.96	45.70 ± 12.89	47.30 ± 12.51	46.26 ± 12.37	<0.001
Sex						<0.001
Male	255,715 (54.6%)	49,885 (42.6%)	56,965 (48.6%)	68,841 (58.8%)	80,024 (68.3%)	
Female	212,843 (45.4%)	67,255 (57.4%)	60,174 (51.4%)	48,298 (41.2%)	37,116 (31.7%)	
SBP (mmHg)	130.25 ± 17.88	122.24 ± 16.34	128.15 ± 17.15	132.91 ± 17.18	137.71 ± 17.09	<0.001
DBP (mmHg)	79.92 ± 10.96	75.10 ± 9.71	78.32 ± 10.32	81.30 ± 10.48	84.96 ± 10.80	<0.001
ALT (U/L)	24.54 ± 22.54	14.86 ± 10.98	19.16 ± 15.04	25.05 ± 18.57	39.09 ± 31.71	<0.001
AST (U/L)	20.91 ± 12.94	18.48 ± 13.22	19.46 ± 11.58	20.96 ± 10.91	24.74 ± 14.80	<0.001
UA (*μ*mol/L)	341.67 ± 92.92	301.99 ± 79.40	324.92 ± 85.05	352.57 ± 88.76	387.21 ± 95.27	<0.001
FPG (mmol/L)	5.26 ± 1.17	4.82 ± 0.49	5.04 ± 0.60	5.26 ± 0.80	5.93 ± 1.88	<0.001
BMI (kg/m^2^)	24.10 ± 4.79	20.44 ± 1.56	23.03 ± 1.30	24.99 ± 1.50	27.93 ± 7.44	<0.001
GGT (U/L)	28.21 ± 36.75	16.63 ± 23.79	21.50 ± 25.49	29.33 ± 33.81	45.39 ± 50.66	<0.001
TG (mmol/L)	1.61 ± 1.31	0.95 ± 0.42	1.25 ± 0.60	1.64 ± 0.83	2.60 ± 2.03	<0.001
LDL(mmol/L)	2.63 ± 0.78	2.40 ± 0.69	2.62 ± 0.74	2.73 ± 0.77	2.76 ± 0.85	<0.001
HTN	162,858 (34.8%)	18,869 (16.1%)	32,680 (27.9%)	47,193 (40.3%)	64,116 (54.7%)	<0.001
DM	27,887 (6.0%)	1,118 (1.0%)	2,702 (2.3%)	5,823 (5.0%)	18,244 (15.6%)	<0.001
HUA	90,521 (19.3%)	9,270 (7.9%)	15,812 (13.5%)	25,051 (21.4%)	40,388 (34.5%)	<0.001
HTN-HUA	44,225 (9.4%)	2,817 (2.4%)	6,216 (5.3%)	12,009 (10.3%)	23,183 (19.8%)	<0.001
Drinking status						<0.001
0	349,834 (74.7%)	97,242 (83.0%)	91,665 (78.3%)	84,146 (71.8%)	76,781 (65.5%)	
1	62,265 (13.3%)	10,571 (9.0%)	13,521 (11.5%)	17,457 (14.9%)	20,716 (17.7%)	
2	35,564 (7.6%)	6,186 (5.3%)	7,995 (6.8%)	9,958 (8.5%)	11,425 (9.8%)	
3	20,895 (4.5%)	3,141 (2.7%)	3,958 (3.4%)	5,578 (4.8%)	8,218 (7.0%)	
Smoking status						<0.001
0	372,055 (79.4%)	99,092 (84.6%)	97,726 (83.4%)	91,973 (78.5%)	83,264 (71.1%)	
1	13,814 (2.9%)	2,580 (2.2%)	3,036 (2.6%)	3,806 (3.2%)	4,392 (3.7%)	
2	6,242 (1.3%)	1,146 (1.0%)	1,360 (1.2%)	1,700 (1.5%)	2,036 (1.7%)	
3	76,447 (16.3%)	14,322 (12.2%)	15,017 (12.8%)	19,660 (16.8%)	27,448 (23.4%)	

Continuous data were expressed as mean ± standard deviation (x¯ ± s), with intergroup differences evaluated by the Kruskal–Wallis H test. Categorical data were presented as counts (percentages) and examined using the chi-square (*χ*^2^) test.

SBP, systolic blood pressure; DBP, diastolic blood pressure; BMI, body mass index; TG, triglyceride; LDL, low-density lipoprotein cholesterol; FPG, fasting plasma glucose; ALT, alanine aminotransferase; GGT, gamma-glutamyltransferase; AST, aspartate aminotransferase; UA, uric acid; HTN-HUA, hypertension plus hyperuricemia.

## Results

### Nonlinear associations of the ZJU index with HTN-HUA, HTN, and HUA: an RCS-based analysis

After adjustment for age, sex, smoking, drinking, GGT, and LDL, ZJU was associated with HTN-HUA, HTN, and HUA (*P* for overall association < 0.001 for each outcome). Separate likelihood-ratio tests also supported nonlinearity for all three outcomes (*P* for nonlinearity < 0.001 for each outcome; Figure 2).

**Figure 2:**
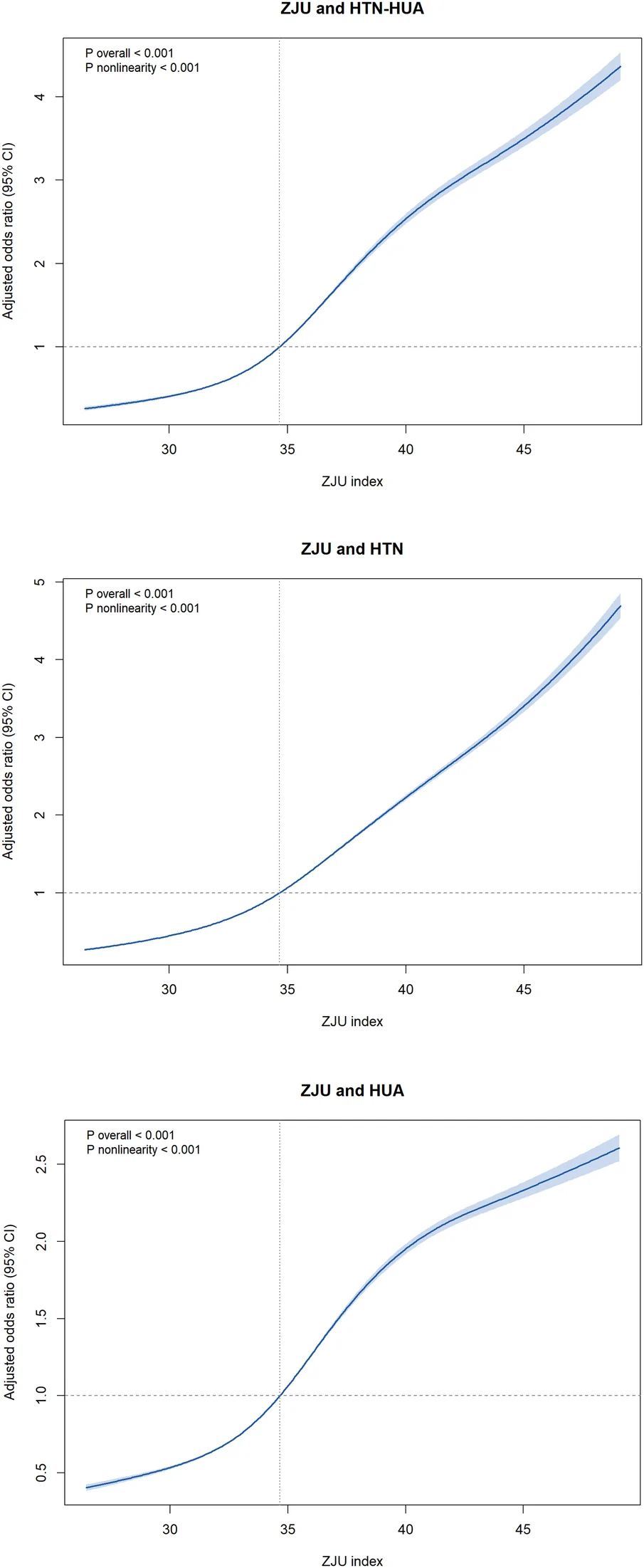
Adjusted RCS associations of ZJU with HTN-HUA, HTN, and HUA. Solid curves show odds ratios relative to ZJU = 34.66; shaded regions indicate 95% confidence intervals. Four knots were placed at ZJU = 28.48, 32.98, 36.50, and 43.78. Models adjusted for age, sex, smoking, drinking, GGT, and LDL. For readability, the displayed range is the 1st–99th percentile of ZJU.

### Baseline characteristics

Table 1 summarizes the baseline characteristics of participants by quartiles of the ZJU index (Q1–Q4). The complete-case sample included 468,558 participants, whose mean age was 45.31 ± 13.10 years. Among them, 212,843 (45.4%) were female. The prevalence of hypertension (HTN) was 34.8% (162,858 cases), hyperuricemia (HUA) was 19.3% (90,521 cases), and comorbid HTN and HUA (HTN-HUA) was 9.4% (44,225 cases). As the ZJU index quartile increased, there was a stepwise elevation in BMI, FPG, ALT, AST, GGT, LDL, and TG levels, alongside a rising prevalence of DM, HTN-HUA, HTN, and HUA. Specifically, relative to the lowest quartile (Q1), the prevalence of HTN rose from 16.1% to 54.7%, HUA from 7.9% to 34.5%, and HTN-HUA from 2.4% to 19.8% across quartiles.

### Logistic regression analysis of ZJU index associations with HTN-HUA, HTN, and HUA

The results of multivariable logistic regression analyses are shown in Table 2. After adjustment for age, sex, smoking, drinking, GGT, and LDL, the odds of HTN-HUA were progressively higher in Q2 through Q4 than in Q1, with OR (95% CI) values of 1.95 (1.86–2.04), 3.40 (3.26–3.55), 7.01 (6.72–7.31), respectively. Corresponding estimates were 1.77 (1.73–1.81), 2.84 (2.78–2.91), 5.66 (5.53–5.79) for HTN and 1.62 (1.57–1.66), 2.39 (2.32–2.45), 4.01 (3.90–4.11) for HUA. All comparisons had *P* < 0.001. The Q4-versus-Q1 OR was numerically larger for HTN-HUA than for HTN or HUA individually.

**Table 2:** Multivariable logistic model estimates of the associations of ZJU index with HTN-HUA, HTN, and HUA.

	Model 1		Model 2		Model 3	
Characteristic	OR(95%CI)	*P*-value	OR(95%CI)	*P*-value	OR(95%CI)	*P*-value
HTN-HUA						
Q1	Ref.		Ref.		Ref.	
Q2	2.27 (2.17–2.38)	<0.001	2.05 (1.96–2.15)	<0.001	1.95 (1.86–2.04)	<0.001
Q3	4.64 (4.45–4.83)	<0.001	3.72 (3.56–3.88)	<0.001	3.40 (3.26–3.55)	<0.001
Q4	10.01 (9.62–10.42)	<0.001	8.06 (7.74–8.40)	<0.001	7.01 (6.72–7.31)	<0.001
HTN						
Q1	Ref.		Ref.		Ref.	
Q2	2.02 (1.97–2.06)	<0.001	1.84 (1.80–1.88)	<0.001	1.77 (1.73–1.81)	<0.001
Q3	3.51 (3.45–3.58)	<0.001	3.03 (2.96–3.09)	<0.001	2.84 (2.78–2.91)	<0.001
Q4	6.30 (6.18–6.42)	<0.001	6.27 (6.13–6.41)	<0.001	5.66 (5.53–5.79)	<0.001
HUA						
Q1	Ref.		Ref.		Ref.	
Q2	1.82 (1.77–1.87)	<0.001	1.71 (1.66–1.75)	<0.001	1.62 (1.57–1.66)	<0.001
Q3	3.17 (3.09–3.25)	<0.001	2.61 (2.54–2.68)	<0.001	2.39 (2.32–2.45)	<0.001
Q4	6.12 (5.98–6.27)	<0.001	4.59 (4.47–4.71)	<0.001	4.01 (3.90–4.11)	<0.001

Model 1 contained no covariate adjustments; Model 2: Adjusted for age and sex; Model 3: Adjusted for age, sex, smoking, drinking, GGT, LDL.

### Subgroup analysis

Subgroup analyses were stratified by age, sex, smoking, drinking, and diabetes status (Table 3). Q4 was associated with higher odds of HTN-HUA than Q1 in every subgroup (all *P* < 0.001). Associations differed across strata of each stratifying variable (all *P* for interaction < 0.001); these interaction tests assess heterogeneity rather than significance within individual subgroups.

**Table 3:** Subgroup analyses of the association between ZJU index and hypertension plus hyperuricemia.

Subgroup	Q1	Q2	Q3	Q4	*P* for interaction
	OR (95%CI)	OR (95%CI)	OR (95%CI)	OR (95%CI)	
Age					<0.001
<40	Ref.	2.26 (2.01–2.55)**	4.51 (4.04–5.03)**	11.56 (10.40–12.84)**	
40–65	Ref.	2.06 (1.93–2.19)**	3.62 (3.41–3.84)**	6.70 (6.33–7.10)**	
>65	Ref.	1.57 (1.43–1.71)**	2.16 (1.98–2.35)**	3.03 (2.77–3.30)**	
Sex					<0.001
Male	Ref.	1.91 (1.82–2.00)**	3.21 (3.06–3.35)**	6.12 (5.86–6.40)**	
Female	Ref.	2.05 (1.72–2.44)**	4.21 (3.58–4.94)**	12.50 (10.71–14.59)**	
Diabetes					<0.001
No	Ref.	1.97 (1.88–2.06)**	3.54 (3.38–3.70)**	8.01 (7.67–8.36)**	
Yes	Ref.	1.59 (1.24–2.04)**	1.99 (1.57–2.51)**	3.21 (2.57–4.03)**	
Smoking					<0.001
0	Ref.	2.00 (1.89–2.11)**	3.57 (3.39–3.76)**	7.51 (7.13–7.90)**	
1	Ref.	1.85 (1.49–2.30)**	3.31 (2.70–4.04)**	6.24 (5.13–7.59)**	
2	Ref.	1.32 (0.96–1.81)	2.04 (1.52–2.72)**	4.08 (3.09–5.40)**	
3	Ref.	1.90 (1.73–2.09)**	3.08 (2.83–3.36)**	6.10 (5.62–6.62)**	
Drinking					<0.001
0	Ref.	2.03 (1.91–2.16)**	3.61 (3.40–3.82)**	8.08 (7.64–8.54)**	
1	Ref.	1.79 (1.62–1.98)**	3.17 (2.90–3.48)**	5.81 (5.32–6.36)**	
2	Ref.	1.85 (1.61–2.13)**	3.20 (2.82–3.65)**	6.11 (5.38–6.93)**	
3	Ref.	1.83 (1.56–2.15)**	2.65 (2.29–3.07)**	4.87 (4.23–5.61)**	

Models adjusted for age, sex, smoking, drinking, GGT, and LDL, omitting the stratifying variable; age was omitted in age-stratified models. *P* for interaction was obtained from likelihood-ratio tests.

** *P* < 0.001.

### Discrimination of HTN-HUA, HTN, and HUA by ZJU and clinical covariate models

Table 4 and Figure 3 compare apparent discrimination in the same 468,558 complete cases. For HTN-HUA, the AUC was 0.725 (95% CI: 0.723–0.727) for ZJU alone, 0.785 (95% CI: 0.783–0.787) for clinical covariates without ZJU, and 0.827 (95% CI: 0.825–0.828) for clinical covariates plus ZJU. The ZJU-alone Youden threshold was 35.66, with sensitivity 72.3% and specificity 61.6%. For HTN, the corresponding AUCs were 0.689, 0.786, and 0.816; for HUA, they were 0.688, 0.780, and 0.804. Adding ZJU increased AUC by 0.041, 0.030, and 0.023 for HTN-HUA, HTN, and HUA, respectively (all paired DeLong *P* < 0.001).

**Table 4:** Apparent ROC performance of ZJU alone and clinical covariate models.

Outcome	AUC	95% CI	Threshold	Specificity	Sensitivity
ZJU alone					
HTN-HUA	0.725	0.723–0.727	35.66	0.616	0.723
HTN	0.689	0.687–0.691	34.64	0.596	0.685
HUA	0.688	0.686–0.690	35.59	0.633	0.650
Clinical covariates					
HTN-HUA	0.785	0.783–0.787	0.09	0.599	0.833
HTN	0.786	0.785–0.787	0.32	0.669	0.758
HUA	0.780	0.779–0.782	0.18	0.541	0.912
Clinical covariates + ZJU					
HTN-HUA	0.827	0.825–0.828	0.09	0.680	0.827
HTN	0.816	0.814–0.817	0.33	0.700	0.778
HUA	0.804	0.802–0.805	0.21	0.634	0.839

Clinical covariates were age, sex, smoking, drinking, GGT, and LDL. The combined model added ZJU as a continuous linear term. ZJU-alone thresholds are ZJU scores.

**Figure 3:**
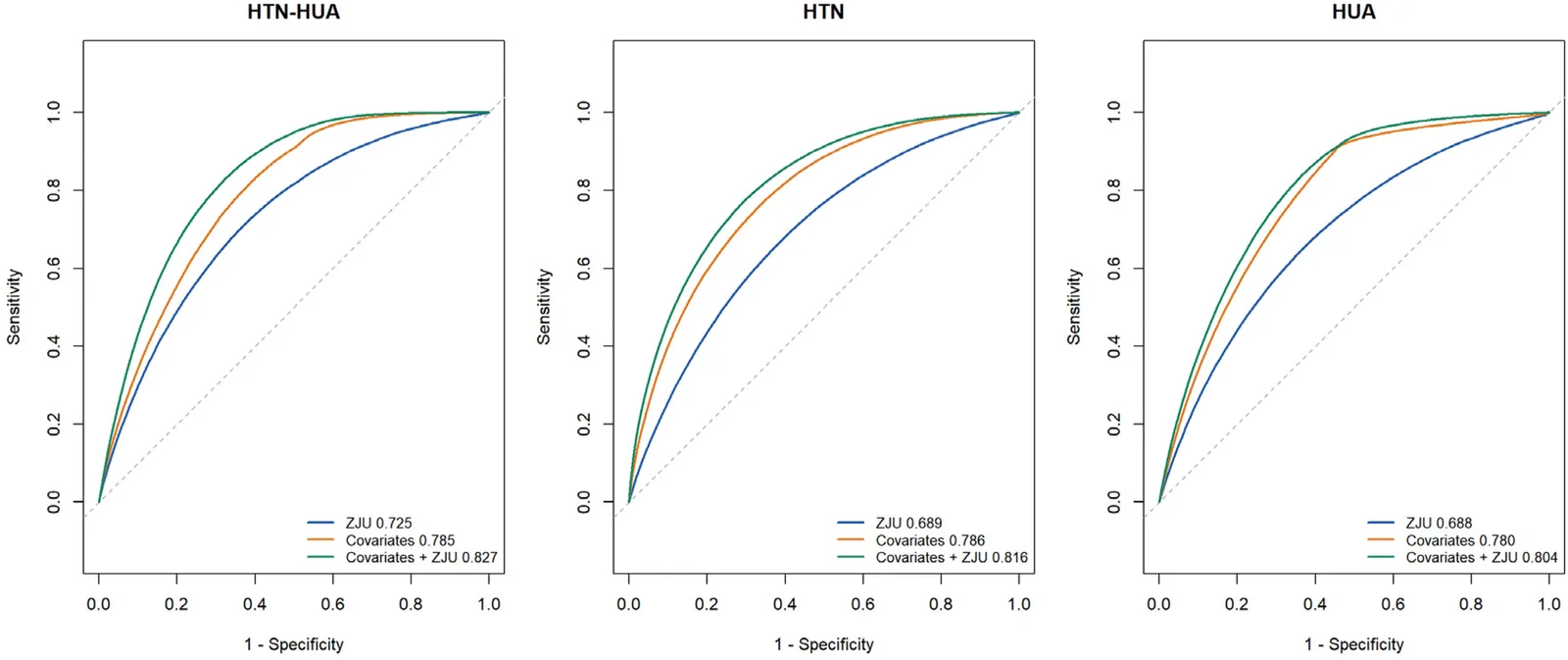
ROC curves for ZJU and clinical covariate models. Curves compare ZJU alone, clinical covariates without ZJU, and clinical covariates plus ZJU. Multivariable curves use fitted probabilities; AUCs represent apparent discrimination in the fitting sample.

The discrimination of ZJU alone for HTN-HUA varied across age and sex strata. The highest age-stratum AUC was observed among participants younger than 40 years: 0.824 (95% CI: 0.820–0.828), with a Youden cut-off of 36.00. The age-specific AUCs were 0.704 in participants aged 40–65 years and 0.586 in those aged >65 years. The AUC was 0.805 (95% CI: 0.798–0.811) in women and 0.678 (95% CI: 0.675–0.681) in men (Figure 4).

**Figure 4:**
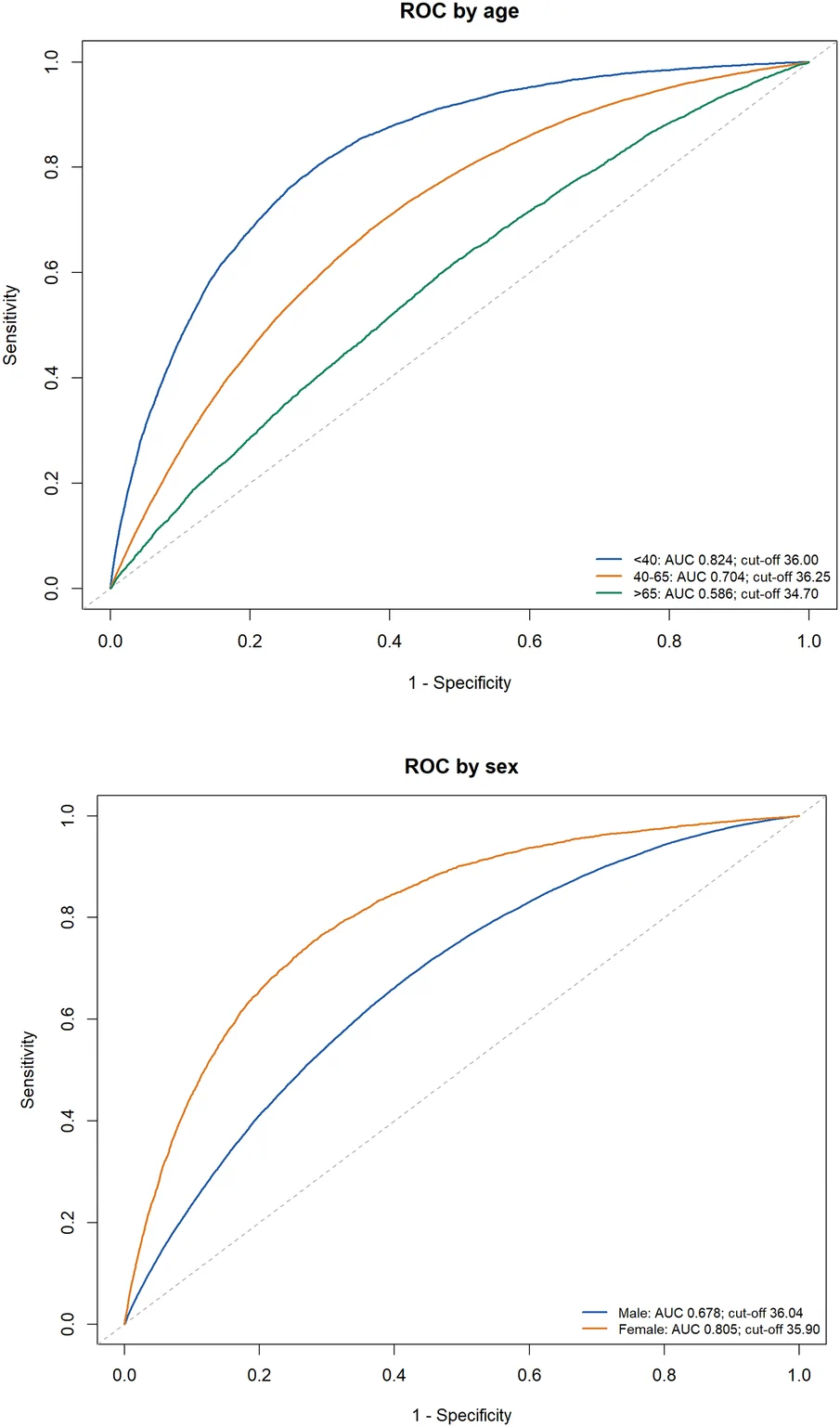
ROC curves of ZJU for HTN-HUA by age and sex.

## Discussion

To our knowledge, this is one of the largest studies to examine the association between the ZJU index and the coexistence of HTN and HUA. Our results reveal that higher ZJU index values are significantly associated with higher odds of prevalent HTN-HUA, as well as HTN and HUA individually, among adults undergoing health examinations at the First Affiliated Hospital of Wannan Medical College in Wuhu, China. Moreover, a graded association was evident when comparing across increasing ZJU quartiles.

Beyond its links to HUA and HTN, the ZJU index showed significant associations with several unfavorable lifestyle behaviors—including smoking and alcohol intake—alongside a range of biochemical markers. As documented in Table 1, progressive elevations in blood pressure (both systolic and diastolic), BMI, FPG, ALT, GGT, LDL, and TG were observed with each ascending ZJU quartile. These observations align closely with prior cross-sectional and prospective cohort reports (31–33). After multivariable adjustment, participants in the highest ZJU quartile had 7.01 times the odds of HTN-HUA compared with those in the lowest quartile (OR: 7.01, 95% CI: 6.72–7.31). Meanwhile, the RCS analysis disclosed a nonlinear association of the ZJU index with the odds of prevalent HTN-HUA. For HTN-HUA, the AUC increased from 0.785 for the clinical covariate model to 0.827 after adding ZJU. Subgroup analyses further indicated that the ZJU index-HTN-HUA association proved to be stronger among younger individuals and females.

The observed heterogeneity across age and sex strata deserves further consideration. The stronger association between the ZJU index and HTN-HUA among younger adults and women may indicate that metabolic abnormalities captured by the ZJU index become particularly informative before the development of more advanced age-related cardiometabolic disease. However, the mechanisms underlying this heterogeneity remain uncertain. Differences in body-fat distribution, hormonal status, lifestyle factors, or baseline cardiometabolic risk may contribute to the observed effect modification, but these explanations could not be directly evaluated in the present dataset. Future prospective studies incorporating detailed assessments of sex hormones, body composition, and lifestyle factors are warranted to clarify these findings. The apparent discrimination of ZJU alone was substantially poorer among participants aged >65 years (AUC, 0.586) than among younger participants. This indicates limited ability to distinguish individuals with and without prevalent HTN-HUA in the oldest age group, despite the observed association. Differences in comorbidity, treatment exposure, and age-related physiology may contribute to this pattern, but these explanations could not be evaluated directly in the present study.

As an index originally developed for identifying NAFLD in Chinese adults, the ZJU index was initially developed by combining BMI, fasting plasma glucose (FPG), triglycerides (TG), alanine aminotransferase (ALT), and aspartate aminotransferase (AST) (20). Furthermore, dyslipidemia, elevated blood pressure, and glycemic abnormalities commonly cluster in metabolic syndrome (34). In this context, because the ZJU index combines anthropometric, glycemic, lipid, and liver-enzyme measures, it may serve as a composite indicator of metabolic status. In a cross-sectional investigation, Wang and colleagues from Zhejiang University demonstrated that the ZJU index achieved an AUC of 0.896 when detecting hepatic steatosis, a finding subsequently corroborated by pathological validation (20). Emerging evidence suggests a link between NAFLD and disorders of lipid and glucose metabolism, as well as dysfunction of adipose tissue (35), and elevated ALT to AST ratios are commonly observed in populations with metabolic dysregulation (36, 37). Therefore, the ZJU index may reflect, to some extent, the overall status of hepatic metabolism.

Importantly, the clinical relevance of the ZJU index may extend beyond hepatic steatosis. Previous evidence has shown that the ZJU index is associated with insulin resistance in the Chinese general population, suggesting that it may reflect broader systemic metabolic dysfunction (23). More recent population-based evidence has also demonstrated significant associations between the ZJU index and hypertension, further supporting its potential value as an indicator of cardiometabolic risk (24). These findings provide a reasonable basis for extending the application of the ZJU index from its original hepatic context toward the assessment of other metabolic and cardiovascular disorders.

In our research, we revealed a nonlinear relationship linking the ZJU index to HTN-HUA comorbidity. Notably, this association was markedly stronger than those between the ZJU index and hypertension alone or hyperuricemia alone. The stronger association observed for HTN-HUA than for HTN or HUA alone may reflect the cumulative effects of interconnected metabolic abnormalities. In a cross-sectional study among patients with hypertension in southern China, BMI and FPG were identified as factors associated with dyslipidemia (38). This finding provides context for the coexistence of adiposity-, glucose-, and lipid-related abnormalities in hypertensive populations. HTN and HUA also share pathways involving renal urate handling and vascular function (12, 25). Because the ZJU index incorporates BMI, FPG, TG, and the ALT/AST ratio, it may capture anthropometric, glycemic, lipid, and liver-enzyme-related features simultaneously. This conceptual overlap may help explain the stronger observed association between the ZJU index and HTN-HUA.

The underlying mechanism for this relationship may involve obesity and insulin resistance. Insulin resistance may contribute to both hypertension and hyperuricemia through alterations in renal sodium and uric acid handling (12). Meanwhile, elevated uric acid may promote endothelial dysfunction through oxidative stress and reduced nitric oxide bioavailability, potentially contributing to vascular dysfunction and blood pressure elevation (12, 25). Taken together, these interconnected pathways provide a plausible biological explanation for the coexistence of hypertension and hyperuricemia in individuals with an unfavorable metabolic profile. TG and FPG are individual components of the ZJU index. The triglyceride-glucose (TyG) index, which combines fasting TG and FPG, is commonly used as a surrogate marker of insulin resistance (39, 40). The link between insulin resistance and the incidence of HTN has been confirmed in numerous studies (41). Furthermore, a large cross-sectional investigation led by Fang et al. reported that TG levels were positively associated with hyperuricemia (42). A plausible mechanistic explanation is that elevated TG, by disturbing free fatty acid metabolism and accelerating adenosine triphosphate breakdown, subsequently increases uric acid generation (43, 44). This mechanistic hypothesis receives support from a prospective cohort investigation by Chien et al., which documented a link between uric acid overproduction and purine metabolism in the triglyceride synthetic pathway (45), thereby indirectly corroborating the findings of our study. Accordingly, the ZJU index may reflect metabolic characteristics associated with prevalent hypertension, hyperuricemia, and their comorbidity.

Our findings also extend recent evidence linking the ZJU index to hyperuricemia. A recent cross-sectional study of 1,772 adults with type 2 diabetes in China reported a positive association between the ZJU index and hyperuricemia, with a graded increase in hyperuricemia prevalence across increasing ZJU categories (26). However, that study focused on patients with type 2 diabetes, whereas our study included a substantially larger population undergoing routine health examinations and specifically examined the coexistence of hypertension and hyperuricemia. Thus, our findings extend the evidence on associations of the ZJU index with prevalent hyperuricemia to the combined phenotype of concurrent hypertension and hyperuricemia.

In previous research, the traditional body mass index has demonstrated high sensitivity to metabolic abnormalities and can reflect metabolic status to some extent, yet it has limitations. After stratification by age and sex, notable heterogeneity was observed in the strength of the association between the ZJU index and HTN-HUA. The association was particularly pronounced among younger adults and women. Sex-specific findings should be interpreted in light of the ZJU formula, which includes an additional 2 points for women. Consequently, the same ZJU quartile boundaries correspond to different thresholds for the other components of the index in men and women, which may influence comparisons across sex strata. This finding may suggest that the ZJU index could capture metabolically adverse phenotypes that are not fully reflected by traditional measures of adiposity alone. However, the underlying mechanisms remain unclear because detailed information on body-fat distribution, hormonal status, and other potentially relevant factors was unavailable in the present dataset. Therefore, these subgroup findings should be interpreted cautiously and require confirmation in prospective studies.

The ZJU index integrates anthropometric and biochemical measures, but whether it has prospective predictive value or clinical utility requires further investigation.

## Strengths and limitations

The present study has several strengths. First, the extremely large sample size of 468,558 adults provided substantial statistical power to characterize the association between the ZJU index and HTN-HUA and enabled detailed subgroup analyses. Second, the use of restricted cubic spline modeling allowed us to evaluate potential nonlinear associations rather than assuming a simple linear relationship. Third, we examined not only the combined HTN-HUA phenotype but also HTN and HUA individually, which facilitated a more comprehensive assessment of the association between the ZJU index and related metabolic outcomes. Finally, the consistency of the association across multiple demographic and behavioral subgroups supports the robustness of the findings.

Several limitations should also be acknowledged. First, the cross-sectional design prevents determination of temporal sequence and causality. Therefore, the observed associations should not be interpreted as evidence that a higher ZJU index directly causes HTN-HUA. Second, despite adjustment for several important confounders, residual confounding cannot be excluded because information on dietary intake, physical activity, socioeconomic status, family history, renal function, and some medication exposures was unavailable or incompletely documented. In particular, medications that influence blood pressure, uric acid metabolism, glucose metabolism, or lipid levels may have affected the observed associations. Third, complete-case analysis may introduce selection bias if data completeness is related to the exposure, outcome, or their determinants. Participants attended health examinations at a single medical center rather than being selected through population-based sampling. The sample should not be considered representative of all adults in Wuhu, and the findings may not be generalizable to people who do not attend health examinations or to other settings. Finally, the records were collected during 2017–2023. Potential changes in health examination attendance, clinical practice, and treatment patterns during this period were not specifically evaluated and may limit applicability to current populations. Prospective studies and independent validation are needed to assess temporal relationships and potential clinical utility.

## Conclusion

Elevated ZJU index values were significantly associated with higher odds of prevalent HTN-HUA among adults undergoing health examinations in Wuhu, China, suggesting its potential value as a marker of the metabolic profile associated with concurrent hypertension and hyperuricemia.

## Data Availability

The raw data supporting the conclusions of this article will be made available by the authors, without undue reservation.
